# The peer reviewers

**DOI:** 10.4103/0970-1591.65418

**Published:** 2010

**Authors:** Sourabh Aggarwal

**Affiliations:** University College of Medical Sciences, New Delhi, India

Dear Editor,

I read the editorial article ‘Peer reviewers - The gate keepers of Science’[1] with due interest. The peer reviewers are indeed the silent backstage workers who help to bring out the refi ned version of the research papers. They are strong pillars who contribute immensely to research papers and good quality scientifi c journals. I sincerely applaud the efforts which went in organizing the workshop for peer reviewers to train them to evaluate manuscripts, besides other things.

Not only do the peer reviewers have to have in depth knowledge of the subject concerned, they should also be trained to identify ethical misconduct in manuscript submission and report the misconduct to the concerned authority at the earliest so that appropriate action is taken. They need to identify various unethical conducts including plagiarism, double publication, fabricated data and falsifying, idea stealing, self plagiarism, copyright infringement. These should be discouraged at all levels and authors promoting it repeatedly should be reported to higher authorities.

The evolution of medical practice stems from the data obtained from the research studies and the scientific journals. Thus, more such workshops should be organized to contribute to good quality of research papers and journals and thus to the evolution of medical practice in a larger perspective.
